# SNA – a toolbox for the stoichiometric analysis of metabolic networks

**DOI:** 10.1186/1471-2105-7-129

**Published:** 2006-03-13

**Authors:** Robert Urbanczik

**Affiliations:** 1 Institute of Pharmacology, University of Bern, Friedbühlstr. 49, CH-3010 Bern, Switzerland

## Abstract

**Background:**

Despite recent algorithmic and conceptual progress, the stoichiometric network analysis of large metabolic models remains a computationally challenging problem.

**Results:**

SNA is a interactive, high performance toolbox for analysing the possible steady state behaviour of metabolic networks by computing the generating and elementary vectors of their flux and conversions cones. It also supports analysing the steady states by linear programming. The toolbox is implemented mainly in Mathematica and returns numerically exact results. It is available under an open source license from: .

**Conclusion:**

Thanks to its performance and modular design, SNA is demonstrably useful in analysing genome scale metabolic networks. Further, the integration into Mathematica provides a very flexible environment for the subsequent analysis and interpretation of the results.

## Background

Advances in genomics have enabled the large scale reconstruction of biochemical reaction networks where the main information provided concerns the stoichiometry and reversibility of the involved reactions. While such models disregard the kinetics of the reactions, biologically meaningful predictions can nevertheless be obtained by analysing the stoichiometrically viable steady states of the network [1]. The aim of SNA is to provide a comprehensive interactive environment for such stoichiometric network analysis.

In mathematical terms the stoichiometrically viable steady states form a convex polyhedral cone which is called the flux cone. Recently [2] it has been pointed out that instead of analysing the full flux cone, one can consider a simpler object, the conversion cone. This amounts to giving a black box description of the metabolism which only takes into account the consumption and production of external compounds. So, the conversion cone describes the overall reactions, (in the sense of [3]) which can be effected in steady state between the external metabolites of the network, disregarding the internal mechanism.

Linear Programming, as in Flux Balance Analysis [4], can be used to search for specific points in these cones. But executing a linear program will just return a single point in the cone, providing only limited information about the whole range of possible steady states the biochemical reaction network can assume. More complete descriptions are obtained by computing a minimal generating set for each cone, i.e. a minimal subset such that each vector in the cone can be represented as a linear combination, with non-negative scalar coefficients, of the subset vectors. If all reactions in the network are irreversible, the flux and the conversion cone are pointed and computing their minimal generating sets amounts to listing the edges of the two cones. In the presence of reversible reactions, however, the description provided by a minimal generating set is less satisfactory. On one hand the cones then may no longer be pointed and many quite different minimal generating sets then exist. On the other hand, if there a reversible reactions, cancellation can occur when combining the vectors of a minimal generating set. So, from the fact that, e.g., each vector in the minimal generating set of the flux cone has a non-zero flow through a specific reversible reaction, one cannot conclude that this reaction must run in any steady state. Hence, in presence of reversible reactions, one may wish to precompute all possible cancellations. This leads to the problem of enumerating all the elementary vectors [2,5] of the flux and of the conversion cone. In the case of the flux cone cone, an elementary vector represents a minimal stoichiometrically viable pathway through the network and is often called an elementary mode [6].

By computing the set of elementary flux vectors a complete and very explicit description of the possible steady state behaviour is obtained and the answers to many questions about the metabolism, such as the maximal yield of some compound and the reactions essential for its synthesis are immediately found by inspecting this set. However, for large networks, enumerating all elementary fluxes may not be desirable, or even feasible, since their number can be very large. It is then often useful to first consider the conversion cone which is much simpler than the flux cone when many elementary fluxes are equivalent with regard to the consumed and produced compounds, differing only in the details of the internal synthesis. While this provides a less complete description of the metabolism than the flux cone, many interesting questions can nevertheless be answered by computing the elementary conversions. Cases in point are (a) the determination of minimal media [2] and (b) checking whether the metabolic model is consistent with an experimentally observed overall reaction and gauging the effciency of the latter [7].

## Implementation

I shall only consider the architecture of the toolbox here since the main mathematical and computational concepts implemented in SNA have been described elsewhere. In particular the Nullspace algorithm used by SNA for calculating elementary fluxes, which runs significantly faster than previous procedures [6], is described in [8]. Further, the concept of a conversion cone is introduced in [2], together with the procedure for computing elementary conversions.

The user interface for SNA is Mathematica and the functionality described in the preceeding section is provided by a Mathematica package called SNAsym. The matrix level computational geometry routines underpinning the operation of SNAsym are delegated to the lower level package SNAmat. SNAmat may be of independent interest to people wishing to avoid using SNA proper and only interested in a computational engine for calculating elementary modes from a stoichiometry matrix.

In contrast to SNAsym, which is pure Mathematica code, the lower level package is a hybrid consisting of Mathematica code communicating with a binary program compiled from Matlab and C sources. The division of labour is that the Mathematica part of SNAmat does the arithmetic, whereas, for speed, the purely logical operation are done by the binary. An important motivation for this arises from the fact that in mathematical terms only rational operations are required in the network analysis tasks implemented by SNA, i.e. no irrational functions such a root taking are needed. Hence, if the stoichiometric factors in the networks are represented by fractions the entire analysis can only lead to numbers which are rational as well and thus have an exact numerical representation. SNA exploits this, by doing all of the arithmetic in Mathematica and using the built-in functionality of this software, to always provides numerically exact results. Due to the division of labour between Mathematica and binary code, SNA is nevertheless fast enough to handle large scale problems. For instance, it takes only 7 minutes to calculate the 5 × 10^5 ^elementary fluxes for the model of the central carbon metabolism of *E. coli *[9] on a state of the art PC.

## Results

Biochemical reactions are represented in SNA by Mathematica expressions such as

adpm+3 hc+pim⇀ atpm+2 hm+h20mco2Xte⇋ co2c.
 MathType@MTEF@5@5@+=feaafiart1ev1aaatCvAUfKttLearuWrP9MDH5MBPbIqV92AaeXatLxBI9gBaebbnrfifHhDYfgasaacH8akY=wiFfYdH8Gipec8Eeeu0xXdbba9frFj0=OqFfea0dXdd9vqai=hGuQ8kuc9pgc9s8qqaq=dirpe0xb9q8qiLsFr0=vr0=vr0dc8meaabaqaciaacaGaaeqabaqabeGadaaakeaafaqabeGabaaabaGaeeyyaeMaeeizaqMaeeiCaa3aaWbaaSqabeaacqqGTbqBaaGccqGHRaWkcqaIZaWmcqqGGaaicqqGObaAdaahaaWcbeqaaiabbogaJbaakiabgUcaRiabbchaWjabbMgaPnaaCaaaleqabaGaeeyBa0gaamrr1ngBPrwtHrhAXaqeguuDJXwAKbstHrhAG8KBLbaceaGccqWFGaaicqWFahITcqqGGaaicqqGHbqycqqG0baDcqqGWbaCdaahaaWcbeqaaiabb2gaTbaakiabgUcaRiabikdaYiabbccaGiabbIgaOnaaCaaaleqabaGaeeyBa0gaaOGaey4kaSIaeeiAaGMaeGOmaiJaeGimaaZaaWbaaSqabeaacqqGTbqBaaaakeaacqqGJbWycqqGVbWBcqqGYaGmdaqhaaWcbaGaeeiwaGLaeeiDaqhabaGaeeyzaugaaOGae8hiaaIae83YHCSaeeiiaaIaee4yamMaee4Ba8MaeeOmaiZaaWbaaSqabeaacqqGJbWyaaGccqGGUaGlaaaaaa@6CB9@

Each metabolite has a body which is a string denoting the chemical species (e.g. adp) and a superscript designating the compartment. Above the strings 'm', 'c', and 'e' are used for mitochondrial, cytosolic and, respectively, extra-cellular metabolites. In addition each metabolite has a rôle, the default rôle being internal while other rôles are specified by a subscript. The subscript *Xt *denotes an external metabolite which can be used both as an input and an output to the network, whereas metabolites with subscript *Xtin *or *Xtout *are restricted to being inputs or, respectively, outputs. Note that rôle and compartment are treated as quite distinct concepts. The reason is that in a study focusing on just an organelle, such as mitochondria, one might wish to treat all cytosolic metabolites as external.

### Functionality

Based on a list of such reactions and an appropriate naming scheme for the reactions, SNA is used to construct the abstract data type *mnet *(metabolic net). The toolbox provides a large set of functions for the basic manipulation of *mnet*'s: e.g. joining them, extracting subnetworks and changing the rôle of the metabolites in the network. Less basic functions include, determining which reactions are feasible, i.e. admit a non-zero steady state flow, or simplifying the network in a way that preserves e.g. the conversion cone.

**Figure 1:**
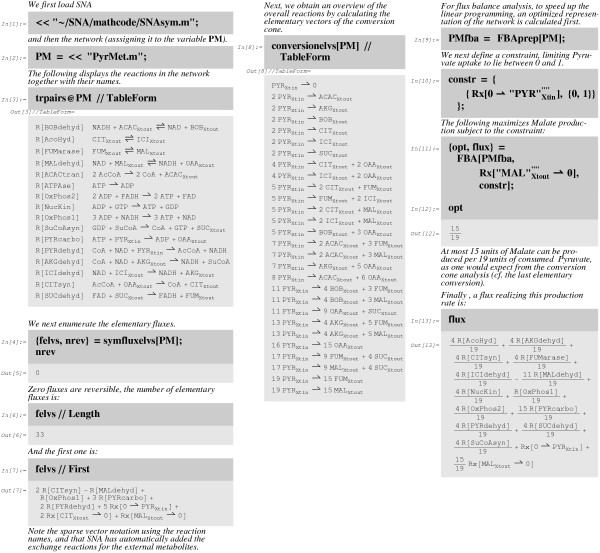
A Mathematica session using SNA to analyse a model of the Pyruvate metabolism in rat liver mitochondria. The model is adapted from [7] where the full metabolite names are given, as well as the explanation for the unusual conversion PYR_Xtin _⇀ 0.

But, of course, the main functionality of SNA is related to the tasks mentioned in the first section. Besides supporting flux balance analysis, the toolbox provides functions analysing the flux or the conversion cone by calculating a minimal generating set or enumerating all the elementary vectors. A session, demonstrating some of the functions of SNA, is shown in Fig. 1.

### Documentation

The main documentation for SNA consists of 9 tutorial Mathematica notebooks. After a basic introduction to the toolbox, the tutorials quickly move on to show how SNA can be used to analyse quite large metabolic networks, considering as examples models of the human cardiac mitochondria [10], the central carbon mechanism of *E. coli *[9] and, finally, a genome-scale model of *S. cerevisiae *[11]. In addition, two of the tutorials demonstrate how metabolic networks can be imported into SNA from external formats. The first shows how to construct an *mnet *given a table of numbers representing a stoichiometry matrix. The second shows how to parse textual representations of metabolic networks such as the ones used by the Palsson group. e.g. [10,11]. Further, SBML models can be read into Mathematica using the MathSBML software developed by B.E. Shapiro [12].

## Conclusion

Currently, the most widely used program for calculating elementary flux vectors probably is Metatool [13]. The latest version of Metatool (5.0) uses the same basic approach [8] as SNA, and the two are also quite similar in terms of required computing time. However, in contrast to Metatool, SNA does not rely on machine precision and always produces numerically exact results. This can be of advantage when analysing large metabolic networks where the stoichiometric factors can span many orders of magnitude, e.g. 7 orders of magnitude for the above mentioned *S. cerevisiae*. Further, compared to Metatool, SNA has as much expanded functionality and, in particular, provides functions for calculating elementary conversions. Finally, the design of SNA is very modular. While this may make the learning curve steeper, it provides the flexibility required to tackle the computational challenge still posed by the analysis of large metabolic networks.

## Availability and requirements

SNA is distributed under an open source license and can be downloaded from . The toolbox runs under PC-Linux and additionally requires Mathematica (5.0 or higher). Further, if one wants to recompile the binary program mentioned in the implementation section, Matlab 6.5 is needed.
